# Supplementary material for: Carprofen Permeation Test through Porcine Ex Vivo Mucous Membranes and Ophthalmic Tissues for Tolerability Assessments: Validation and Histological Study
Source: Vet Sci. 2020 Oct 10;7(4):152. doi: 10.3390/vetsci7040152 (PMC7711503; doi:10.3390/vetsci7040152)
Supplement: Supplementary file 1 [file vetsci-07-00152-s001.docx]

**Supplementary material**


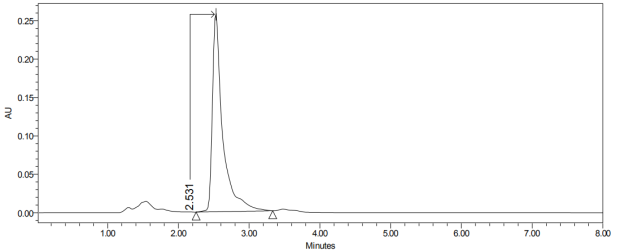

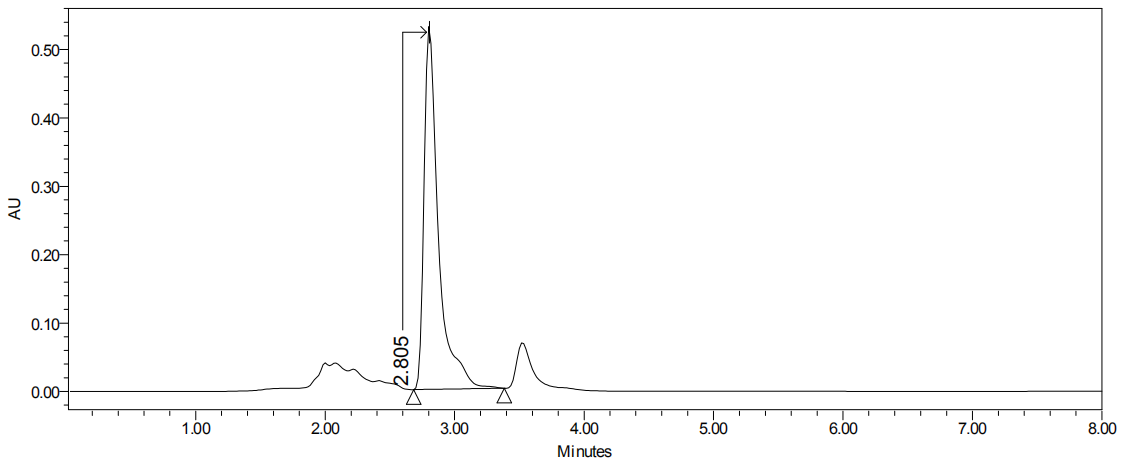


**Figure S1.** PBSo ( PBS saturated with ophthalmic tissue) spiked with Carprofen (top) and receptor solution after 6h of conjunctiva permeation (bottom). Y-axis in the figure is expressed in arbitrary units (AU).


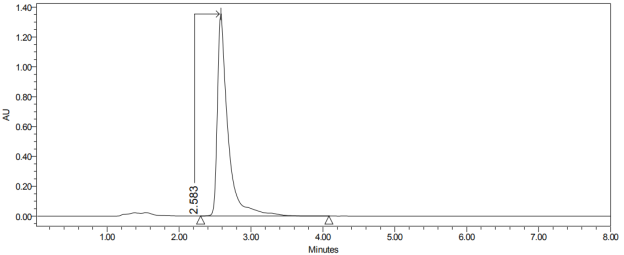

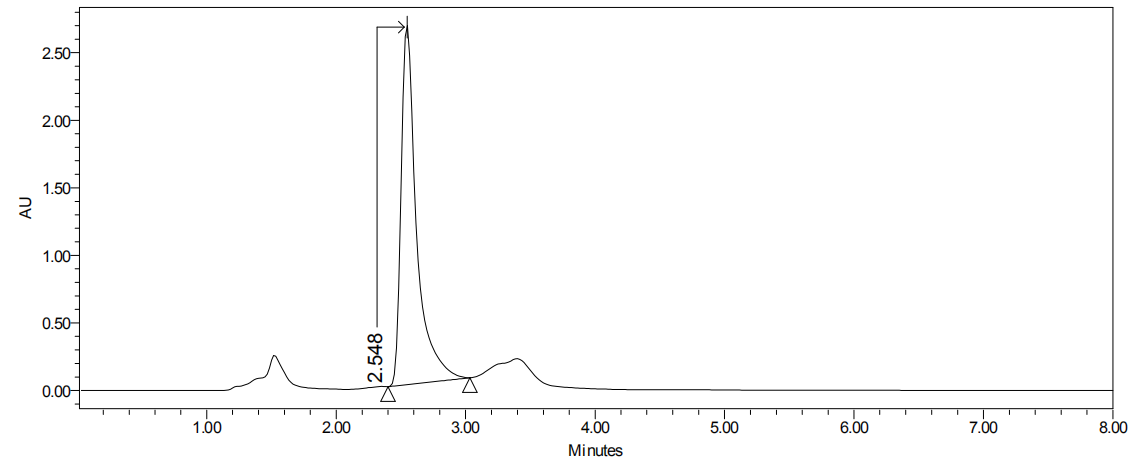


**Figure S2.** PBSo spiked with Carprofen (top) and receptor solution after 6h of cornea permeation (bottom). Y-axis in the figure is expressed in arbitrary units (AU).


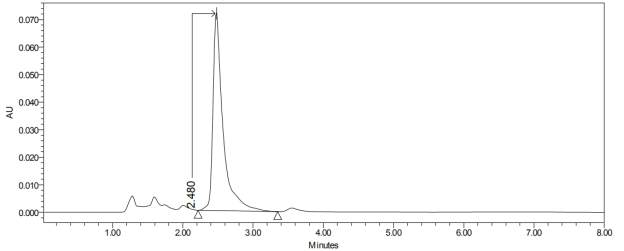

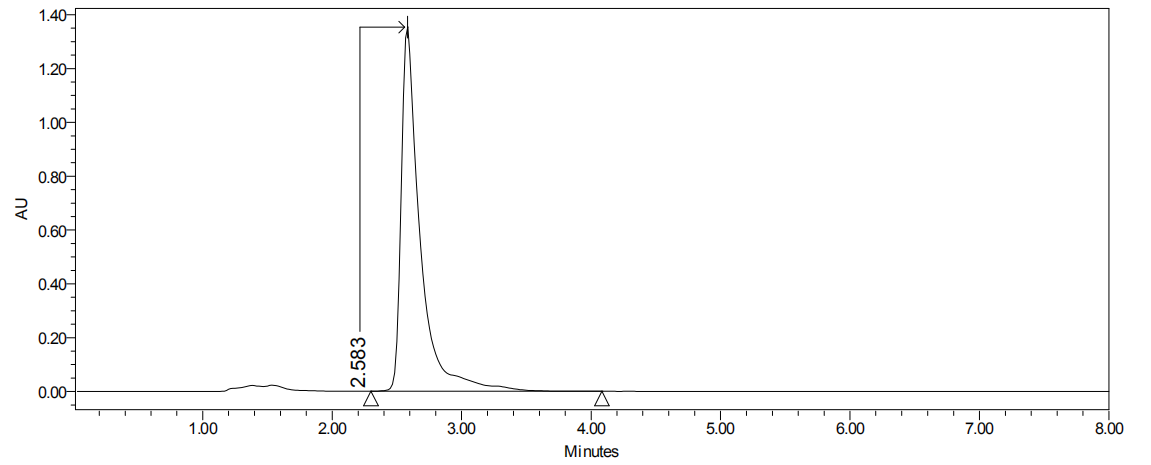


**Figure S3.** PBSo spiked with Carprofen (top) and receptor solution after 6h of sclera permeation (bottom). Y-axis in the figure is expressed in arbitrary units (AU).


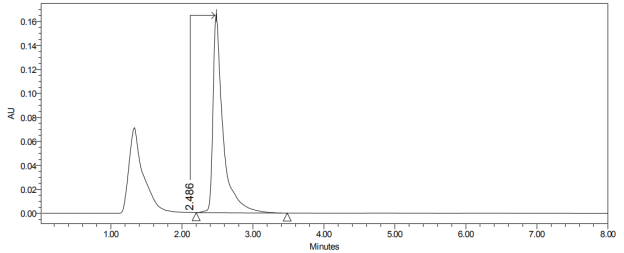

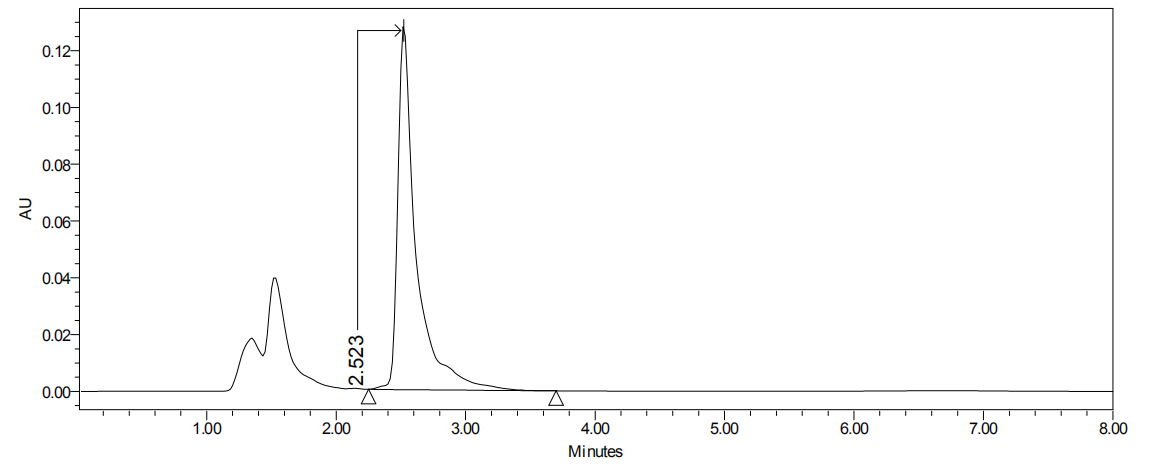


**Figure S4.** PBSm (PBS saturated with mucous) spiked with Carprofen (top) and receptor solution after 6h of buccal permeation (bottom). Y-axis in the figure is expressed in arbitrary units (AU).


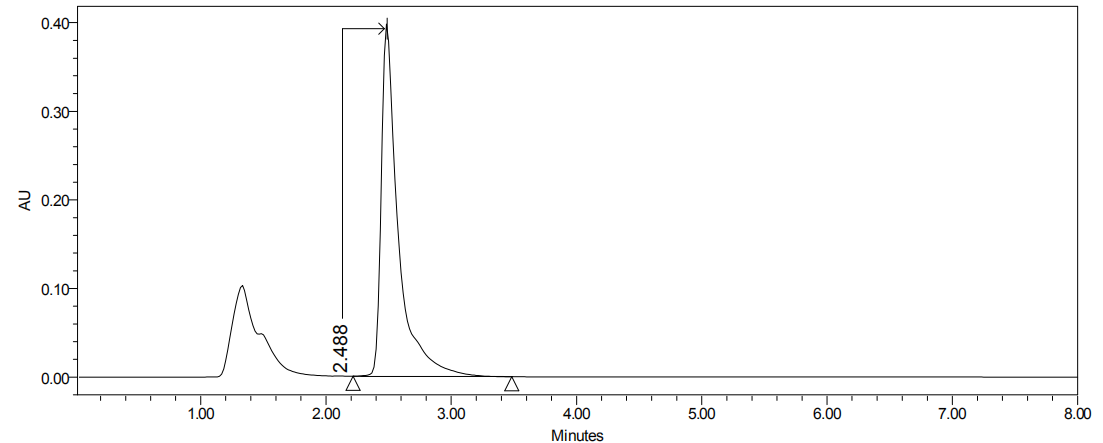

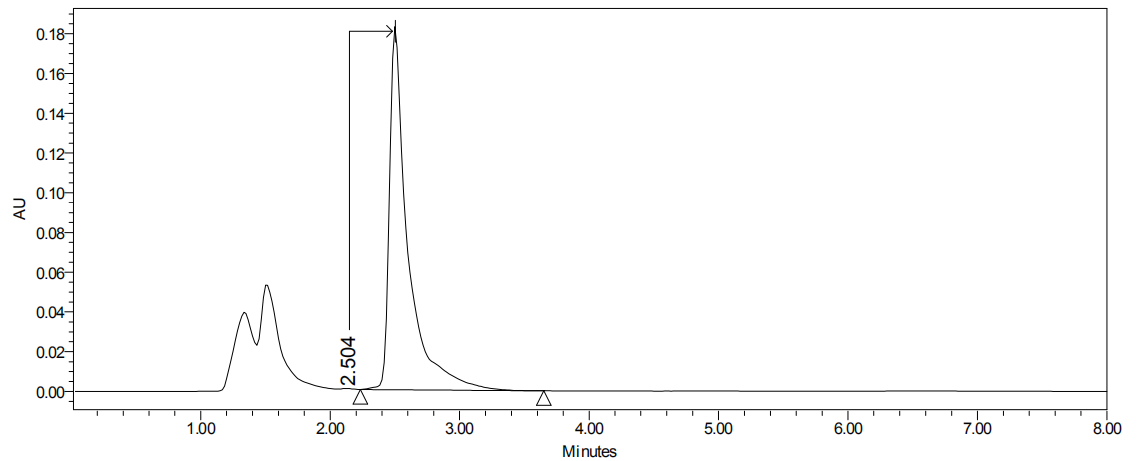


**Figure S5.** PBSm spiked with Carprofen (top) and receptor solution after 6h of sublingual permeation (bottom). Y-axis in the figure is expressed in arbitrary units (AU).

**Table S1.** Six calibration curves of Carprofen (high range) expressed by concentration (µg/mL) versus arbitrary units (AU). Linearity test about ratios of AU/Concentration. ANOVA test and P Value.

| **High range** |  |  |  |  |  |  |  |  |
| --- | --- | --- | --- | --- | --- | --- | --- | --- |
| **Concentration (µg/mL)** | **Curve 1 (AU)** | **Curve 2 (AU)** | **Curve 3 (AU)** | **Curve 4 (AU)** | **Curve 5 (AU)** | **Curve 6 (AU)** | **Ratios (AU/Concentration)** | **Value** |
| 100 | 16205860 | 16202720 | 16055210 | 17112110 | 17931100 | 17560670 | Table Analysed |  |
| 50 | 8055477 | 8743008 | 8230302 | 8526197 | 8903436 | 8757483 | Parameter |  |
| 25 | 4016840 | 4254043 | 4033393 | 4276343 | 4179592 | 4167502 | One-way analysis |  |
| 12,5 | 2061085 | 2129517 | 1973098 | 2092546 | 2143851 | 2092321 | of variance |  |
| 6,25 | 1005324 | 1070250 | 999881 | 1018093 | 1070961 | 1059305 | **P value** | **0,404** |
| 3,125 | 553937 | 570976 | 514336 | 521232 | 534325 | 531480 | **P value summary** | **NS** |

**Table S2.** Six calibration curves of Carprofen (low range) expressed by concentration (µg/mL) versus arbitrary units (AU). Linearity test about ratios of AU/Concentration. ANOVA test and P Value.

| **Low range** |  |  |  |  |  |  |  |  |
| --- | --- | --- | --- | --- | --- | --- | --- | --- |
| **Concentration (µg/mL)** | **Curve 1** | **Curve 2** | **Curve 3** | **Curve 4** | **Curve 5** | **Curve 6** | **Ratios (AU/Concentration)** | **Value** |
| 6.25 | 1005324 | 1070250 | 999881 | 1018093 | 1070961 | 1059305 | Table Analysed |  |
| 3.125 | 553937 | 570976 | 514336 | 521232 | 534325 | 531480 | Parameter |  |
| 1.5625 | 276803 | 285296 | 262066 | 254689 | 263846 | 267145 | One-way analysis |  |
| 0.7813 | 164322 | 164520 | 135319 | 127661 | 133578 | 139190 | of variance |  |
|  |  |  |  |  |  |  | **P value** | **0.0783** |
|  |  |  |  |  |  |  | **P value summary** | **NS** |

**Table S3.** Slopes and standard deviation (desvest.) of Y-intercept about six Carprofen calibration curves (low range) needed to calculate Determination Limit and Quantification Limit (expressed by mean and desvest.).

|  | **Curve 1** | **Curve 2** | **Curve 3** | **Curve 4** | **Curve 5** | **Curve 6** | **Parameter** | **Mean** |
| --- | --- | --- | --- | --- | --- | --- | --- | --- |
| **Variables** |  |  |  |  |  |  |  | µg/mL |
| Slope | 154700 | 166500 | 157900 | 163000 | 171700 | 168500 | Determination Limit | 0,17 ± 0.13 |
| Desvest. of Y-intercept | 16980 | 11720 | 4203 | 6317 | 1671 | 1674 | Quantification Limit | 0,51 ± 0.40 |

**Table S4.** Experimental concentrations of six Carprofen calibration curves (high range in µg/mL) and its corresponding mean, standard deviation (desvest.), relative error (RE %) and relative standard derivation (RSD %).

| **High range** |  |  |  |  |  |  |  |  |  |  |
| --- | --- | --- | --- | --- | --- | --- | --- | --- | --- | --- |
| **Concentration (µg/mL)** | **Exp. Curve 1** | **Exp. Curve 2** | **Exp. Curve 3** | **Exp. Curve 4** | **Exp. Curve 5** | **Exp. Curve 6** | **Mean** | **Desvest.** | **RE (%)** | **RSD (%)** |
| 100 | 100,150 | 98,666 | 99,619 | 100,000 | 100,176 | 100,145 | 99,793 | 0,590 | 0,21 | 0,59 |
| 50 | 49,764 | 52,795 | 50,900 | 49,956 | 50,137 | 50,107 | 50,610 | 1,138 | -1,22 | 2,25 |
| 25 | 24,779 | 25,181 | 24,919 | 25,150 | 23,863 | 24,133 | 24,671 | 0,549 | 1,32 | 2,22 |
| 12,5 | 12,679 | 12,112 | 12,164 | 12,404 | 12,540 | 12,389 | 12,381 | 0,216 | 0,95 | 1,75 |
| 6,25 | 6,148 | 5,596 | 6,139 | 6,132 | 6,572 | 6,544 | 6,189 | 0,355 | 0,98 | 5,74 |
| 3,125 | 3,355 | 2,525 | 3,134 | 3,232 | 3,587 | 3,557 | 3,232 | 0,389 | -3,42 | 12,04 |

**Table S5.** Experimental concentrations of six Carprofen calibration curves (low range in µg/mL) and its corresponding mean, standard deviation (desvest.), relative error (RE %) and relative standard derivation (RSD %).

| **Low range** |  |  |  |  |  |  |  |  |  |  |
| --- | --- | --- | --- | --- | --- | --- | --- | --- | --- | --- |
| **Concentration (µg/mL)** | **Exp. Curve 1** | **Exp. Curve 2** | **Exp. Curve 3** | **Exp. Curve 4** | **Exp. Curve 5** | **Exp. Curve 6** | **Mean** | **Desvest.** | **RE (%)** | **RSD (%)** |
| 6,25 | 6,195 | 6,218 | 6,235 | 6,229 | 6,251 | 6,253 | 6,230 | 0,022 | 0,31 | 0,35 |
| 3,125 | 3,278 | 3,219 | 3,160 | 3,180 | 3,126 | 3,121 | 3,181 | 0,060 | -1,78 | 1,88 |
| 1,5625 | 1,486 | 1,503 | 1,563 | 1,545 | 1,550 | 1,552 | 1,533 | 0,031 | 1,87 | 2,01 |
| 0,7813 | 0,759 | 0,778 | 0,761 | 0,765 | 0,791 | 0,793 | 0,775 | 0,015 | 0,87 | 1,95 |
